# Effects of the Quantum Vacuum at a Cosmic Scale and of Dark Energy

**DOI:** 10.3390/e26121042

**Published:** 2024-11-30

**Authors:** Emilio Santos

**Affiliations:** Departamento de Física, Universidad de Cantabria, 39005 Santander, Spain; santose@unican.es

**Keywords:** dark energy, quantum vacuum, quantized Einstein equation

## Abstract

The Einstein equation in a semiclassical approximation is applied to a spherical region of the universe, with the stress-energy tensor consisting of the mass density and pressure of the ΛCDM cosmological model plus an additional contribution due to the quantum vacuum. Expanding the equation in powers of Newton constant G, the vacuum contributes to second order. The result is that at least a part of the acceleration in the expansion of the universe may be due to the quantum vacuum fluctuations.

## 1. Dark Energy, Cosmological Constant, and Vacuum Fluctuations

In this paper, I will study the possible effects of the quantum vacuum at a scale larger than the typical distances between galaxies. The work may provide clues to ascertain whether vacuum fluctuations might be the origin of dark energy.

The hypothesis of dark energy (DE) has been introduced in order to explain the *accelerating* expansion of the universe [1,2,3,4]. DE consists of a density and pressure

(1) $$\rho_{DE}=-\left(1+\varepsilon \right)p_{DE}≃\left(6.0\pm 0.2\right)\times 10^{-27}\;\mathrm{kg}/m^{3},$$

filling space homogeneously [5,6]. The nature of dark energy is unknown, but it is an empirical fact that $\left|\varepsilon \right|<<1$ shows that its effect is fairly equivalent to a cosmological constant [7].

As is well known, the cosmological constant (CC) was introduced by Einstein in order to obtain a stationary (although not stable) model of the universe. Later on, the discovery of the expansion of the universe made the CC useless, but it was a recurrent possibility for about 70 years although without too much empirical support [8]. The view changed in 1998, when it was discovered that the expansion of the universe was accelerating [1,2], which might be seen as the effect of a CC, although the less committed assumption has been made that the acceleration is caused by a hypothetical ingredient named DE. In any case, some of the proposals about the nature of DE are similar to previous assumptions about the origin of a possible CC.

An early proposal was that CC may correspond to the energy and pressure of the quantum vacuum. If this was the case, a plausible assumption seemed to be that its value could be obtained via a combination of the universal constants *c*, *ħ*, *G*. There is a unique combination with dimensions of density, that is, Planck density with a value

(2) $$\rho_{Planck}=\frac{c^{4}}{G^{2}\hbar }≃10^{97}\;\mathrm{kg}/m^{3},$$

which is about 123 orders greater than either the known value of the DE Equation (1) or any reasonable value for a CC. This big discrepancy has been named the “cosmological constant problem” [8,9]. Many proposals have been made in the past for the origin of a CC [8] (or DE, see, e.g., [10] and references therein) that shall not be discussed here. One of them has been the quantum vacuum origin, as said above. If this is the case, then some mechanism should exist reducing Equation (2) to Equation (1), but the fine tuning required looks unplausible, even conspiratory [8]. However, I point out that, although the mean energy of the vacuum might be canceled by some mechanism, the fluctuations cannot cancel it completely, which suggests that the fluctuations could give rise to an effective CC or DE.

Using dimensional arguments, any theory aimed at explaining the DE Equation (1) would involve at least a new parameter, in addition to the universal constants, c, ħ, G. If we choose the parameter to be a mass, *m*, then the value of dark energy could be written in the form (with c=1)

(3) $$\rho_{DE}\approx \frac{G^{n}m^{2n+4}}{\hbar^{n+3}},$$

with *n* being a real number. The choice n=−2 would remove *m* and lead to the Planck density Equation (2), but n=1 may give the observed value Equation (1) for $\rho_{DE}$ provided that *m* is of order the pion mass. Indeed, more than forty years ago Zeldovich [11] proposed a formula like Equation (3) with n=1 in order to obtain a plausible value for a cosmological constant. Furthermore, he interpreted the result in terms of the mass *m* and its associated “Compton wavelength” λ, as follows:

(4) $$\Lambda \equiv \rho_{CC}∼-\frac{Gm^{2}}{\lambda }\times \frac{1}{\lambda^{3}},\lambda \equiv \frac{\hbar }{m}.$$

Thus, Equation (4) looks like the energy density corresponding to the (Newtonian) gravitational energy of two particles of mass *m* placed at a distance λ, assuming that such an energy appears in every volume $\lambda^{3}$ (although in Equation (4), the gravitational energy Λ would be negative if both masses were positive). Zeldovich’s interpretation was that the “particles” were actually vacuum fluctuations. Hence, his hypothesis that a finite CC might exist derived from the fluctuations of the quantum vacuum. In recent times, some modifications of Zeldovich’s proposal have been attempted as an explanation for dark energy, identifying the CC with the DE Equation (1) [12].

In the present paper, I again study the possibility that vacuum fluctuations produce a gravitational effect similar to a DE. Unlike previous papers [12], where heuristic arguments were used, here I will use a more formal quantum approach to the vacuum fluctuations. Indeed, the quantum vacuum fluctuations are specific quantum features; therefore, classical equations like Friedman’s (see next section) are not appropriate in order to obtain the contents of the universe from the observable value of the accelerated expansion. In summary, a correct approach should involve quantum field theory and general relativity.

We should deal with quantized general relativity, but no fully satisfactory quantum gravity is available. Thus, I will follow an approximate or *effective* approach to the gravity of a quantum system, that is, the quantum vacuum. In fact, I will integrate a semi-classical Einstein equation of general relativity approximated to the second order in the Newton constant *G*, as will be explained in Section 3.2. An effective treatment of the quantum vacuum will be studied in Section 3.1.

## 2. Revisiting the Argument for Dark Energy

Our quantum approach in Section 3 will parallel the standard procedure to obtain the DE Equation (1) from the observed accelerated expansion of the universe [1,2,3]. For this reason, I will revisit that method, which allows one to relate observable properties of spacetime to the contents of the universe via general relativity. Indeed, astronomical observations are compatible with the universe having a Friedmann–Lemaître–Robertson–Walker (FLRW) metric with flat spatial slices [13] of the form

(5) $$ds^{2}=-dt^{2}+a{\left(t\right)}^{2}\left[dr^{2}+r^{2}\left(d\theta^{2}+\sin^{2}\theta d\phi^{2}\right)\right],$$

where the parameter a(t) takes into account the expansion of the universe. Indeed, at the present time $t_{0}$, it is related to the measurable Hubble constant, $H_{0}$, and the deceleration parameter, $q_{0}$, via

(6) $${\left[\frac{\dot{a}}{a}\right]}_{t_{0}}=H_{0},\;{\left[\frac{\ddot{a}}{a}\right]}_{t_{0}}=-H_{0}^{2}q_{0}.$$

From the function a(t), the contents of the universe may be obtained by solving the Friedman equation (which is a particular case of the Einstein equation that is appropriate for the FLRW metric). The result is that, aside from the baryonic mass density, $\rho_{B}$, two hypothetical ingredients seem to exist, namely, an additional (dark) matter having the mass density $\rho_{DM}$ with negligible pressure, and another component with positive energy density, $\rho_{DE},$ but negative pressure $p_{DE}=-\rho_{DE},$ labeled dark energy (DE). In fact, the following relations are obtained, as proved below

(7) $$\begin{array}{ccc} {\left[\frac{\dot{a}}{a}\right]}^{2} & = & \frac{8\pi G}{3}\left(\rho_{B}\left(t\right)+\rho_{DM}\left(t\right)+\rho_{DE}\right),\\ \frac{\ddot{a}}{a} & = & \frac{8\pi G}{3}\left(\frac{1}{2}\left[\rho_{B}\left(t\right)+\rho_{DM}\left(t\right)\right]-\rho_{DE}\right),\; \end{array}$$

where small effects of radiation and matter pressure are neglected.

The baryonic density $\rho_{B}$ is well known from the measured abundances of light chemical elements, which allows for the calculation of $\rho_{DE}$ and $\rho_{DM}$ from the empirical quantities $H_{0}$ and $q_{0}$ via comparison of Equation (7) with Equation (6). The result may be summarized in the ΛCDM model. In it, baryonic matter density, $\rho_{B}$, represents about 4.6% of the matter content, while cold dark matter (CDM) and dark energy (represented by the greek letter Λ) contribute densities $\rho_{DM}∼$ 24% and $\rho_{DE}∼$ 71.3%, respectively. The values obtained by this method agree with data from other observations. For instance, cold dark matter, in an amount compatible with $\rho_{DM}$, is needed in order to explain the observed (almost flat) rotation curves in galaxies.

In this section, I revisit the derived relation of the metric of spacetime, at the cosmological scale, with the mass densities $\rho_{B}\left(t\right),\;\rho_{DM}\left(t\right),\;\rho_{DE}$ and pressure $p_{DE}=-\rho_{DE}$ of the ΛCDM model. The standard approach is to use the FLRW metric as said above, but for our purposes it is more convenient to deal with a metric alternative to FLRW, Equation (5), using curvature coordinates for spherical symmetry whose most general metric is as follows:

(8) $$ds^{2}=g_{rr}\left(r^{\prime },t^{\prime }\right)dr^{\prime 2}+r^{\prime 2}\left(d\theta^{2}+\sin^{2}\theta d\phi^{2}\right)-g_{tt}\left(r^{\prime },t^{\prime }\right)dt^{\prime 2}.$$

This metric may be appropriate for a small enough region of the universe around us but large in comparison with typical distances between galaxies [14].

The relation between the metrics Equations (5) and (8) is as follows. We perform a change of variables in Equation (5), that is,

(9) $$r=a^{-1}r^{\prime },\;t=t^{\prime }-\frac{\dot{a}}{2a}r^{2},\;\dot{a}\equiv \frac{da}{dt},$$

so chosen that, after some algebra, Equation (5) becomes Equation (8) where

(10) $$\begin{array}{ccc} g_{rr}\left(r^{\prime },t^{\prime }\right) & = & 1+{\left(\frac{\dot{a}}{a}\right)}^{2}r^{\prime 2}+O\left(r^{\prime 3}\right),\\ g_{tt}\left(r^{\prime },t^{\prime }\right) & = & 1+\frac{\ddot{a}}{a}r^{\prime 2}+O\left(r^{\prime 3}\right),\ddot{a}\equiv \frac{d^{2}a}{dt^{2}}. \end{array}$$

The calculation may be performed to order $r^{\prime 2}$, consistent with the metric Equation (8) being appropriate for a small region around us.

Now, I shall solve (the classical) Einstein equation for the metric Equation (8) with a stress-energy tensor given by the ΛCDM model as described above, that is, the mass (or energy) density $\rho_{mat}$ of matter may be taken as the sum of two *homogeneous* contributions, that is, a $\rho_{mat}=\rho_{B}\left(t\right)+\rho_{DM}\left(t\right),$ meaning baryonic and dark matter, respectively, with negligible pressure, plus a dark energy with homogeneus density $\rho_{DE}$ and negative pressure $p_{DE}=-\rho_{DE}.$

The metric Equation (8) requires spherical symmetry, that is, both mass density and pressure should depend only on the radial coordinate *r* and time *t*. Obviously, this is not the case for the actual universe where matter is mainly localized in galaxies. In practice, an approximation consists of averaging the mass density over the whole region. I point out that a similar approximation is usually made when the FRW metric Equation (1) is used [14]. The result of solving the Einstein equation for the metric Equation (8) is

(11) $$\begin{array}{ccc} g_{rr} & = & 1+\frac{8\pi G}{3}\left[\rho_{B}+\rho_{DM}+\rho_{DE}\right]r^{2}+O\left(r^{3}\right),\\ g_{tt} & = & 1+\frac{8\pi G}{3}\left[\frac{1}{2}\left[\rho_{B}+\rho_{DM}\right]-\rho_{DE}\right]r^{2}+O\left(r^{3}\right),\; \end{array}$$

as is well known [14]. A comparison of Equation (11) with Equation (10) leads to Equation (7).

Now, I proceed to the proof of Equation (11). I will neglect terms of order $O\left(r^{3}\right)$ and ignore the (slow) change of the metric coefficients with time, a change derived from the slow time dependence of the matter density $\rho_{mat}$. With $g_{rr}=g_{tt}=1$ for r=0, we obtain the following elements for a metric like Equation (8) [15]

(12) $$\begin{array}{ccc} g_{rr}\left(r\right) & = & {\left(1-\frac{2Gm\left(r\right)}{r}\right)}^{-1},\;m\left(r\right)=m_{mat}\left(r\right)+m_{DE}\left(r\right)\\ m_{mat}\left(r\right) & = & \int_{\left|z\right|<r}\rho_{mat}d^{3}z,m_{DE}\left(r\right)\equiv \int_{\left|z\right|<r}\rho_{DE}d^{3}z,\\ g_{tt}\left(r\right) & = & \exp\gamma ,\gamma =2G\int_{\left|x\right|<r}\frac{m\left(x\right)+4\pi x^{3}p_{DE}\left(x\right)}{x^{2}-2Gxm\left(x\right)}dx. \end{array}$$

as typically, $Gm\left(r\right)<<r$, an approximation, is appropriate, consisting of expanding Equation (12) in powers of the Newton constant G, retaining terms up to order $O\left(G^{2}\right)$. For Equation (12), this approximation agrees with order $O\left(r^{2}\right)$ in the radial parameter r, as may be easily checked. Thus, I may write

(13) $$g_{rr}=1+\frac{2Gm\left(r\right)}{r}+\frac{4G^{2}m{\left(r\right)}^{2}}{r^{2}}+O\left(G^{3}\right).$$

(14) $$\begin{array}{ccc} g_{tt} & = & 1+2G\int_{0}^{r}\left(\frac{m\left(x\right)}{x^{2}}+4\pi xp\left(x\right)\right)dx+2G^{2}{\left[\int_{0}^{r}\left(\frac{m\left(x\right)}{x^{2}}+4\pi xp\left(x\right)\right)dx\right]}^{2}\\ & & +4G^{2}\int_{0}^{r}m\left(x\right)\left(\frac{m\left(x\right)}{x^{3}}+4\pi p\left(x\right)\right)dx+O\left(G^{3}\right). \end{array}$$

The terms of order $O\left(G^{2}\right)$ will be relevant when the quantum vacuum is taken into account, as in the quantum approach of the next section but, here we may neglect those terms. As said above, I take the contents of the universe into account as in the ΛCDM model, that is, a (homogeneous) mass density given by $\rho_{B}+\rho_{DM}+\rho_{DE}$ and pressure $p_{DE}=-\rho_{DE}.$ Then, Equations (13) and (14) give Equation (11).

## 3. A Quantum Treatment

The aim of this article is to improve the standard derivation of the contents of the universe from astronomical observations, essentially the Hubble constant and the acceleration parameter as in Equations (5)–(7) This involves a quantized, rather than classical, Einstein equation and the inclusion of the quantum vacuum fluctuations. I shall start dealing with the approach to the quantum vacuum and a semi-classical approximate Einstein equation of general relativity.

### 3.1. The Quantum Vacuum

Vacuum fluctuations are straightforward consequences of quantum field theory (QFT), but their treatment is far from trivial. In a naive approach, the energy of the vacuum is divergent, for instance, in the quantum electromagnetic field the vacuum energy in a finite volume is $E_{vac}=\sum_{j}\hbar \omega_{j}$, which goes to *∞* when we take all possible normal modes *j* into account. There are procedures to avoid the divergence, from the simplistic “normal ordering” rule to the sophisticated renormalization methods in quantum electrodynamics; the latter is extremely successful, as is well known. In the study of the influence of vacuum fluctuations at a cosmic scale, at least two alternatives arise. We may assume that the vacuum energy density is just given by Equation (1), with the associated pressure, thus explaining the nature of dark energy. Another possibility is that the vacuum energy density is strictly zero, with dark energy having a different origin, maybe unrelated to the quantum vacuum. An intermediate possibility is that just a part of DE is due to the quantum vacuum. In any case, it is natural within quantum theory to assume that the vacuum energy is an observable that I should represent by a quantum operator ${\hat{\rho }}_{vac}\left(r,t\right)$. Its vacuum expectation may be either finite (positive), e.g., given by Equation (1), or just zero. In this paper, I attempt to study the latter posibility. Indeed, if it is finite it should be either all or a part of DE, but no further study of that possibility will be made here.

Thus, I shall assume the following

(15) $${\hat{p}}_{vac}\left(r,t\right)=-{\hat{\rho }}_{vac}\left(r,t\right),\langle vac\left|{\hat{\rho }}_{vac}\left(r,t\right)\right|vac\rangle =0,\langle vac\left|{\hat{\rho }}_{vac}{\left(r,t\right)}^{2}\right|vac\rangle >0,$$

where I have included the vacuum pressure operator, the former equality deriving from the requirement of Lorentz invariance, which is plausible. Indeed, we are considering a spacetime very close to Minkowski. The inequality in Equation (15) means that the vacuum energy density fluctuates. I point out that Equation (15) might be derived in principle from quantum field theory, including the vacuum fluctuations of all fields (belonging to the standard model of high energy physics) and their interactions.

For our purposes, Equation (15) is not sufficient in order to characterize the quantum vacuum. The quantities relevant for our work are the two-point correlations of the density and pressure of the vacuum. In an approximate flat (Minkowski) space, the vacuum should be invariant under translations and rotations, whence the vacuum expectation of the product of two vacuum density operators (at equal times) should be a universal function, *C*, of the distance $\left|r_{1}-r_{2}\right|$, that is,

(16) $$\frac{1}{2}\langle vac\left|{\hat{\rho }}_{vac}\left(r_{1}\right)\;{\hat{\rho }}_{vac}\left(r_{2}\right)+\;{\hat{\rho }}_{vac}\left(r_{2}\right){\hat{\rho }}_{vac}\left(r_{1}\right)\right|vac\rangle =C\left(\left|r_{1}-r_{2}\right|\right).$$

The function C may be named the self-correlation of the vacuum energy density.

In this article, I will assume that the integral of C(x) extended over the whole space is nil, that is,

(17) $$\int_{\left|r_{2}\right|\in \left(-\infty ,\infty \right)}C\left(\left|r_{1}-r_{2}\right|\right)d^{3}r_{2}=\int_{\left|r\right|\in \left(-\infty ,\infty \right)}C\left(\left|r\right|\right)d^{3}r=0.$$

I believe that this assumption is plausible once we assume $\langle vac\left|{\hat{\rho }}_{vac}\left(r,t\right)\right|vac\rangle =0$ as in Equation (15). The opposite assumption might be worth studying but will not be made here. The implication

$$\langle vac\left|{\hat{\rho }}_{vac}\left(r,t\right)\right|vac\rangle =0\Rightarrow \int {\hat{\rho }}_{vac}\left(r,t\right)d^{3}r=0,$$

is similar to the ergodic property in the (classical) stochastic processes (See Appendix A for the problems of interpretation).

Equation (17) may be generalized to the self-correlation of the pressure and the cross-correlation of density and pressure to be introduced in Section 3.2.

### 3.2. Semiclassical Approximation to a Quantum Einstein Equation

We must solve the Einstein equation of GR for a stress-energy tensor that should be defined for an essentially quantum system involving the quantum vacuum. However, there is no fully satisfactory quantum gravity theory that unifies GR and quantum mechanics, and we must rely on approximations. The left side of Einstein equation of GR is known only in its classical form, that is, the Einstein tensor $G_{\mu \nu }$. But the right side should be an stress-energy tensor defined in quantum form, that is, an operator ${\hat{T}}_{\mu \nu }$ in the Hilbert space. An equation with a classical tensor on one side and a quantum operator tensor on the other is inconsistent, and the standard solution to the problem is the semiclassical approximation, that is,

(18) $$G_{\mu \nu }=-8\pi G\langle \psi \left|{\hat{T}}_{\mu \nu }\right|\psi \rangle ,$$

where ∣ψ〉 is the quantum state of the system. Then both sides of the (Einstein) equation are c-numbers. The Einstein tensor is a function of the metric elements and their first and second derivatives with respect to the coordinates. Therefore, Equation (18) is a partial differential equation involving the metric elements, which in our case consists of just two non-trivial ones, that is, $g_{rr}$ and $g_{tt},$ see Equation (8).

The stress-energy tensor operator ${\hat{T}}_{\mu \nu }$ may be written, similar to its classical counterpart in Section 2, in terms of energy density and pressure operators. In the classical case, the contributions to the mass (or energy) density, and pressure, are given by the ΛCDM cosmological model. That is, mass densities $\rho_{mat}=\rho_{B}+\rho_{DM}$ and $\rho_{DE}$, and the pressure $p_{DE}=-\rho_{DE}$. In Equation (18), these quantities should be considered expectation values, in the state ∣ψ〉, of appropriate quantum observables, that is,

$$\langle \psi \left|{\hat{\rho }}_{mat}\right|\psi \rangle =\rho_{mat},\langle \psi \left|{\hat{\rho }}_{DE}\right|\psi \rangle =\rho_{DE},\langle \psi \left|{\hat{p}}_{DE}\right|\psi \rangle =p_{DE},$$

so that Equation (18) becomes just like the classical Einstein equation solved in Section 2, if we ignore the possible quantum vacuum contribution.

The semiclassical approximation in Equation (18) presents the problem that any information about fluctuations is lost. Indeed, fluctuations of a quantum observable, say, $\hat{M}$, appear only in the higher moments $\langle \psi \left|{\hat{M}}^{n}\right|\psi \rangle$, n≥2. However, in our case all operators (of either mass density or pressure) appear linearly in the stress-energy tensor ${\hat{T}}_{\mu \nu }$, whence fluctuations are neglected in Equation (18). Thus, in order to study the effect of vacuum fluctuations I propose a different semiclassical approximation, that is, using the *expectation value of an integrated, rather than differential, Einstein equation* defined in Equation (18). That semiclassical approximation may be represented as follows:

(19) $$g_{\mu \nu }=\langle \psi \left|{\hat{g}}_{\mu \nu }\left(\left\{{\hat{T}}_{\lambda \sigma }\right\}\right)\right|\psi \rangle ,$$

where $\left\{g_{\mu \nu }\right\}$ are the elements of the metric and $\left\{{\hat{g}}_{\mu \nu }\right\}$ the corresponding operators in a hypothetical quantum Einstein equation.

Equation (19) would be useless except if we were able to get the function ${\hat{g}}_{\mu \nu }\left(\left\{{\hat{T}}_{\lambda \sigma }\right\}\right)$ of the operators ${\hat{g}}_{\mu \nu }$ in terms of the elements of the operator tensor ${\hat{T}}_{\lambda \sigma }.$ This will be possible just in some extremely simple situations. One of them is our case of the metric Equation (8) with just two non-trivial metric elements and where we may neglect the time dependence of the stress-energy tensor. Then, the two functions ${\hat{g}}_{rr}\left(\left\{{\hat{T}}_{\lambda \sigma }\right\}\right)$ and ${\hat{g}}_{tt}\left(\left\{{\hat{T}}_{\lambda \sigma }\right\}\right)$ will be similar to Equation (12) with two modifications. Firstly, we shall substitute the quantum operators ${\hat{\rho }}_{mat}$, ${\hat{\rho }}_{DE}$, and ${\hat{p}}_{DE}$ for the (classical) expectations $\rho_{mat},\;\rho_{DE}$ and $p_{DE},$ respectively. Secondly, we shall include the vacuum operators ${\hat{\rho }}_{vac}\left(r,t\right)$ and ${\hat{p}}_{vac}\left(r,t\right)$.

The result is the following Equation (19), consisting of just two metric elements written in terms of the quantum operators for energy density and pressure,

(20) $$\begin{array}{ccc} g_{rr}\left(r\right) & = & \langle \psi \left|1+\frac{2G\hat{m}\left(r\right)}{r}+\frac{4G^{2}\hat{m}{\left(r\right)}^{2}}{r^{2}}\right|\psi \rangle +O\left(G^{3}\right),\\ g_{tt}\left(r\right) & = & \langle \psi \left|1+2G\int_{0}^{r}x^{-2}\hat{m}\left(x\right)dx+G^{2}\sum_{n=1}^{5}{\hat{c}}_{n}\right|\psi \rangle +O\left(G^{3}\right), \end{array}$$

where we define

(21) $$\begin{array}{ccc} \;\hat{m}\left(r\right) & = & {\hat{m}}_{mat}\left(r\right)+{\hat{m}}_{DE}\left(r\right)+{\hat{m}}_{vac}\left(r\right),{\hat{m}}_{vac}\left(r\right)\equiv \int_{\left|z\right|\leq r}{\hat{\rho }}_{vac}d^{3}z\\ {\hat{m}}_{mat}\left(r\right) & \equiv & \int_{\left|z\right|\leq r}{\hat{\rho }}_{mat}d^{3}z=\int_{\left|z\right|>r}{\hat{\rho }}_{mat}d^{3}z,{\hat{m}}_{DE}\left(r\right)\equiv \int_{\left|z\right|\leq r}{\hat{\rho }}_{DE}d^{3}z. \end{array}$$

I will label $\left\{{\hat{c}}_{n}\right\}$ the operators corresponding to the terms of the sum Equation (14), which now are promoted to be operators, that is,

(22) $$\begin{array}{ccc} {\hat{c}}_{1} & = & 4\int_{0}^{r}x^{-3}\hat{m}{\left(x\right)}^{2}dx,\\ {\hat{c}}_{2} & = & \int_{0}^{r}x^{-2}dx\int_{0}^{r}y^{-2}dy\left[\hat{m}\left(x\right)\hat{m}\left(y\right)+\hat{m}\left(y\right)\hat{m}\left(x\right)\right],\\ {\hat{c}}_{3} & = & 32\pi^{2}\int_{0}^{r}xdx\int_{0}^{r}ydy\left[\hat{p}\left(x\right)\hat{p}\left(y\right)+\hat{p}\left(y\right)\hat{p}\left(x\right)\right],\\ {\hat{c}}_{4} & = & 8\pi \int_{0}^{r}\left[\hat{m}\left(x\right)\hat{p}\left(x\right)+\hat{p}\left(x\right)\hat{m}\left(x\right)\right]dx,\\ {\hat{c}}_{5} & = & 8\pi \int_{0}^{r}x^{-2}dx\int_{0}^{r}ydy\left[\hat{m}\left(x\right)\hat{p}\left(y\right)+\hat{p}\left(y\right)\hat{m}\left(x\right)\right]. \end{array}$$

In the passage from Equations (13) and (14), consisting of numerical quantities (c-numbers), to Equations (20)–(22) involving operators, the problem appears to involve the ordering of operators that do not commute in general. In our case, there are at most two operators in the products in Equation (22), and they appear in symmetrical order, which is most plausible.

Actually, the solution to Equation (18) presents a difficulty similar to the classical Equations (8) and (12). That is, the solution Equations (20)–(22) are valid only if the energy-momentum tensor operator ${\hat{T}}_{\mu \nu }$ depends on the coordinate *r* but not on the angular coordinates, θ,ϕ. I will solve the problem as in the classical case, Equations (13) and (14), that is, averaging the energy density over large enough regions. However, in the quantum domain the solution is more involved. In fact, in the classical domain the dynamical variables are directly observables while in the quantum domain the dynamical variables are represented by operators (usually labeled “observables”) and the actually observable quantities are the expectation values of the “observables” in the appropriate state ∣ψ〉. Thus, I will assume that the semiclassical Equations (20)–(22) are valid when we deal with regions having dimensions much larger than typical distances between galaxies, as is the case in our work.

The metric element $g_{rr}$ consists of the following two terms:

$$g_{rr}=g_{rr}^{\mathrm{model}}+g_{rr}^{vac},$$

where the superindex “model” stands for the ΛCDM model. The former term will be calculated to order $O\left(G\right)$ because the contribution of order $O\left(G^{2}\right)$ is negligible (see the comment below Equation (12)). The latter (vacuum term) should be obtained to order $O\left(G^{2}\right)$ because the term of order $O\left(G\right)$ is nil, see Equation (15). Then, for $O\left(G\right)$ we obtain

(23) $$\begin{array}{ccc} g_{rr} & = & 1+\frac{2G}{r}\int_{\left|z\right|<r}\left(\langle \psi \left|{\hat{\rho }}_{\mathrm{model}}\left(z\right)\right|\psi \rangle +\langle \psi \left|{\hat{\rho }}_{vac}\left(z\right)\right|\psi \rangle \right)d^{3}z\\ & = & 1+\frac{2G}{r}\int_{\left|z\right|<r}\langle \psi \left|{\hat{\rho }}_{\mathrm{model}}\left(z\right)\right|\psi \rangle d^{3}z, \end{array}$$

which will reproduce the standard result, i.e., the first Equation (11) because that term involves

$$\langle \psi \left|{\hat{\rho }}_{\mathrm{model}}\left(z\right)\right|\psi \rangle =\rho_{\mathrm{model}}=\rho_{B}+\rho_{DM}+\rho_{DE}.$$

Similarly, the expectation of ${\hat{g}}_{tt}$ to order $O\left(G\right)$ will reproduce the second Equation (11).

### 3.3. Contribution of the Quantum Vacuum

Taking Equation (20) into account, the term of order $O\left(G^{2}\right)$ of the $g_{rr}$ metric element is

(24) $$\begin{array}{ccc} g_{rr}^{vac} & = & \frac{4G^{2}}{r^{2}}\langle \psi \left|{\hat{m}}_{vac}{\left(r\right)}^{2}\right|\psi \rangle =\frac{4G^{2}}{r^{2}}\langle \psi \left|{\left[\int_{\left|z\right|<r}{\hat{\rho }}_{vac}\left(z\right)d^{3}z\right]}^{2}\right|\psi \rangle \\ & = & \frac{2G^{2}}{r^{2}}\int_{\left|z\right|<r}d^{3}z\int_{\left|v\right|<r}d^{3}v\langle \psi \left|{\hat{\rho }}_{vac}\left(v\right)\;{\hat{\rho }}_{vac}\left(z\right)+\;{\hat{\rho }}_{vac}\left(z\right){\hat{\rho }}_{vac}\left(v\right)\right|\psi \rangle . \end{array}$$

Generalizing Equation (17), I assume that the two-point correlation function, *C*, depends only on the distance $\left|v-z\right|$, that is,

(25) $$\frac{1}{2}\langle \psi \left|{\hat{\rho }}_{vac}\left(v\right)\;{\hat{\rho }}_{vac}\left(z\right)+\;{\hat{\rho }}_{vac}\left(z\right){\hat{\rho }}_{vac}\left(v\right)\right|\psi \rangle =C\left(\left|v-z\right|\right),$$

which implies in particular that we may neglect the possible perturbations of the vacuum correlations due to the presence of matter. The function $C\left(\left|v-z\right|\right)$ should be obviously positive for small values of $\left|v-z\right|$ and decrease as $\left|v-z\right|$ increases, but as argued in Section 3.1 we are here concerned with the case when the v integral over the whole space is nil, that is,

(26) $$\int C\left(\left|v-z\right|\right)d^{3}v=\langle \psi \left|{\hat{\rho }}_{vac}\left(z\right)\right|\psi \rangle =0,$$

see Equation (17). Therefore, $C\left(\left|v-z\right|\right)$ will be negative for large values of $\left|v-z\right|$. An illustrative example of a function with this behavior is the following:

(27) $$\begin{array}{ccc} C\left(x\right) & = & an^{3}\exp\left(-3nx/b\right)-a\exp\left(-3x/b\right),n>>1\\ & \to & C\left(0\right)=a\left(n^{3}-1\right),C\left(x\right)≃-a\exp\left(-3x/b\right)\;\mathrm{for}\;x>>b, \end{array}$$

involving two parameters $\left\{a,b\right\}$. These parameters measure roughly the size of the fluctuations and the the range of their correlation function. The behavior of the function C(x) suggests introducing an auxiliary function F(x) such that

(28) $$C\left(x\right)=n^{3}F\left(nx\right)-F\left(x\right),$$

where n>>1 is a real number and $F\left(x\right)$ is a positive function of the argument that I assume rapidly decreasing at infinity, that is, fulfilling

(29) $$\lim_{x\to \infty }x^{3}F\left(x\right)=0\Rightarrow \lim_{x\to \infty }x^{3}C\left(x\right)=0.$$

Equation (28) guarantees that Equation (26) holds true. Indeed, for integrals over the whole space we have

(30) $$\int d^{3}xn^{3}F\left(nx\right)=\int d^{3}x^{\prime }F\left(x^{\prime }\right)=\int d^{3}xF\left(x\right)\Rightarrow \int d^{3}xC\left(x\right)=0.$$

Now, we may evaluate Equation (24) by taking Equation (25) into account. I start with the following *v*-integral of $C\left(\left|v-z\right|\right)$

(31) $$I\equiv \int_{\left|v\right|<r}C\left(\left|v-z\right|\right)d^{3}v=n^{3}\int_{\left|v\right|<r}F\left(n\left|v-z\right|\right)d^{3}v-\int_{\left|v\right|<r}F\left(\left|v-z\right|\right)d^{3}v.$$

In the limit n→∞, the function $n^{3}F\left(nx\right)$ becomes proportional to a 3D Dirac’s delta $\delta^{3}\left(x\right)$, as may be shown taking Equation (29) into account. Thus, for very large *n* the relevant contribution to the first integral of Equation (31) comes from the region where $\left|v-z\right|$ is small. Hence, we may extend the *v*-integral to the whole space with fair approximation provided that $\left|z\right|<r$ but neglect it if $\left|z\right|>r.$ That is, we may write

$$n^{3}\int_{\left|v\right|<r}F\left(n\left|v-z\right|\right)d^{3}v≃\Theta \left(r-\left|z\right|\right)n^{3}\int_{\left|v\right|\in \left(0,\infty \right)}F\left(n\left|v-z\right|\right)d^{3}v,$$

where the step function $\Theta \left(y\right)=1$ if y≥0, $\Theta \left(y\right)=0$ otherwise. Hence, Equation (31) gives

$$\begin{array}{ccc} I & ≃ & \Theta \left(r-\left|z\right|\right)n^{3}\int_{\left|v\right|\in \left(0,\infty \right)}F\left(n\left|v-z\right|\right)d^{3}v-\int_{\left|v\right|<r}F\left(\left|v-z\right|\right)d^{3}v\\ & = & \Theta \left(r-z\right)n^{3}\int_{\left|x\right|\in \left(0,\infty \right)}F\left(nx\right)d^{3}x-\int_{\left|v\right|<r}F\left(\left|v-z\right|\right)d^{3}v\\ & = & \Theta \left(r-z\right)\int_{\left|x^{\prime }\right|\in \left(0,\infty \right)}F\left(x^{\prime }\right)d^{3}x^{\prime }-\int_{\left|v\right|<r}F\left(\left|v-z\right|\right)d^{3}v\\ & = & \Theta \left(r-z\right)\int_{\left|v\right|\in \left(0,\infty \right)}F\left(\left|v-z\right|\right)d^{3}v-\int_{\left|v\right|<r}F\left(\left|v-z\right|\right)d^{3}v, \end{array}$$

leading to

(32) $$I=\Theta \left(r-z\right)\int_{\left|v\right|\geq r}F\left(\left|v-z\right|\right)d^{3}v\;-\Theta \left(z-r\right)\int_{\left|v\right|<r}F\left(\left|v-z\right|\right)d^{3}v.$$

It is the case that I will integrate for z≤r everywhere in the rest of this section whence Equation (32) becomes

$$I=\Theta \left(r-z\right)\int_{\left|v\right|\geq r}F\left(\left|v-z\right|\right)d^{3}v\;$$

in the following.

We obtain, taking Equations (24) and (25) into acount,

(33) $$\begin{array}{ccc} J & \equiv & \int_{v\geq r,z<r}C\left(\left|v-z\right|\right)d^{3}vd^{3}z=4\int_{z<r}d^{3}z\int_{v>r}d^{3}vF\left(\left|v-z\right|\right)\\ & = & 32\pi^{2}G^{2}r^{-2}\int_{0}^{r}z^{2}dz\int_{r}^{\infty }v^{2}dv\int_{-1}^{1}duF\left(\sqrt{v^{2}+z^{2}-2vzu}\right), \end{array}$$

where u≡cosθ, θ is the angle between the vectors v and z. We know neither the two-point correlation function $C\left(\left|v-z\right|\right)$ nor $F\left(\left|v-z\right|\right)$ in detail, but I propose to characterize the latter by just two parameters (see Equation (27)), namely, the size *D* and the range γ. That is, I will approximate the angular integral in Equation (33) as follows:

(34) $$f\equiv \int_{-1}^{1}duF\left(\sqrt{v^{2}+z^{2}-2vzu}\right)\approx D\Theta \left(\gamma -\left|v-z\right|\right),$$

where $\Theta \left(x\right)$ is the step function, and we assume that the parameter γ>0 is small in the sense that γ<<r. Thus, we obtain

(35) $$g_{rr}^{vac}≃32\pi^{2}G^{2}r^{-2}D\int_{r-\gamma }^{r}z^{2}dz\int_{r}^{z+\gamma }v^{2}dv.$$

For later convenience, I will summarize the steps going from Equation (33) to Equation (35), writing the following, slightly more general, relation valid for any $\alpha \left(v,z\right),$

(36) $$\int_{v<r,z<r}C\left(\left|v-z\right|\right)\alpha \left(v,z\right)d^{3}vd^{3}z=8\pi^{2}D\int_{r-\gamma }^{r}z^{2}dz\int_{r}^{z+\gamma }\alpha \left(v,z\right)v^{2}dv.$$

The integrals in Equation (35) are trivial and we obtain

(37) $$g_{rr}^{vac}≃\frac{G^{2}}{r^{2}}\times 32\pi^{2}D\int_{r-\gamma }^{r}z^{2}dz\left[\frac{{\left(z+\gamma \right)}^{3}}{3}-\frac{r^{3}}{3}\right]=16\pi^{2}G^{2}D\gamma^{2}r^{2}+O\left(\gamma^{3}\right).$$

The ratio γ/r<<1 is small because γ is a length typical of quantum fluctuations, while *r* is of order the typical distance amongs galaxies (see comment after Equation (8)). Therefore, we may neglect terms of order $\gamma^{3}$ whence we obtain

(38) $$g_{rr}^{vac}≃16\pi^{2}G^{2}Kr^{2},K\equiv D\gamma^{2},$$

where I have substituted the single parameter *K* for the product *D* times $\gamma^{2}$. In the following, I take the constant *K* as the relevant parameter, avoiding any detail about its origin from the two-point correlation of vacuum fluctuations $C\left(\left|v-z\right|\right).$

The terms of order $O\left(G^{2}\right)$ of $g_{tt}$, Equation (22), may be obtained in a way similar to those of $g_{rr}$. For the first term, we obtain

$$\begin{array}{ccc} c_{1} & \equiv & \langle \psi \left|{\hat{c}}_{1}\right|\psi \rangle =4\int_{0}^{r}x^{-3}dx\langle \psi \left|\hat{m}{\left(x\right)}^{2}\right|\psi \rangle \\ & = & 4\int_{0}^{r}x^{-3}dx\int_{0}^{x}d^{3}z\int_{0}^{x}d^{3}vC\left(\left|v-z\right|\right), \end{array}$$

where $C\left(\left|v-z\right|\right)$ is the correlation function Equation (28). I will firstly perform the *x* integral, that is,

$$\begin{array}{ccc} c_{1} & = & 4\int_{z<r}d^{3}z\int_{v<r}d^{3}vC\left(\left|v-z\right|\right)\int_{\max\left(v,z\right)}^{r}x^{-3}dx\\ & = & 2\int_{z<r}d^{3}z\int_{v<r}d^{3}vC\left(\left|v-z\right|\right)\left(\frac{1}{\max{\left(v,z\right)}^{2}}-\frac{1}{r^{2}}\right)\\ & = & 16\pi^{2}Dr^{-2}\int_{r-\gamma }^{r}z^{2}dz\left\{\frac{1}{3}\left[{\left(z+\gamma \right)}^{3}-r^{3}\right]-r^{2}\left(z+\gamma -r\right)\right\}, \end{array}$$

where I have taken Equation (36) into account. The result is that $c_{1}$ is of order $O\left(\gamma^{3}\right)$, whence this term contributes but slightly to $g_{tt}.$

In order to obtain c_2_, I start performing the *x* and *y* integrals, that is,

$$\begin{array}{ccc} c_{2} & \equiv & 8\int_{0}^{r}x^{-2}dx\int_{0}^{r}y^{-2}dy\int_{z<x}d^{3}z\int_{v<y}d^{3}vC\left(\left|v-z\right|\right)\\ & = & \int_{z<r}d^{3}z\int_{v<r}d^{3}vC\left(\left|v-z\right|\right)\int_{z}^{r}x^{-2}dx\int_{v}^{r}y^{-2}dy\\ & = & \int_{z<r}\left(\frac{1}{z}-\frac{1}{r}\right)d^{3}z\int_{v<r}\left(\frac{1}{v}-\frac{1}{r}\right)d^{3}vC\left(\left|v-z\right|\right). \end{array}$$

Taking Equation (36) into account, we obtain

$$\begin{array}{ccc} c_{2} & = & 8\pi^{2}Dr^{-2}\int_{r-\gamma }^{r}z\left(r-z\right)\left[\frac{1}{2}r\left({\left(z+\gamma \right)}^{2}-r^{2}\right)-\frac{1}{3}\left({\left(z+\gamma \right)}^{3}-r^{3}\right)\right]dz\\ & = & 8\pi^{2}Dr^{2}\gamma^{2}+O\left(\gamma^{3}\right)≃8\pi^{2}r^{2}K. \end{array}$$

Now, we must compute the $G^{2}$ contribution to $g_{tt}$ coming from the pressure operator ${\hat{p}}_{vac}\left(r,t\right)$ of the vacuum, that is, the terms $c_{3},\;c_{4}$, and $c_{5}$. Before proceeding, I must deal with a difficulty due to the fact that Equation (12) is just valid for spherical symmetry. Actually, that symmetry holds neither for the distribution of matter in the region of interest nor for the stress-energy of the quantum vacuum. In fact, the stress-energy appears in the form of localized operators of energy density ${\hat{\rho }}_{vac}\left(r,t\right)$ and pressure ${\hat{p}}_{vac}\left(r,t\right)$. Actually, this was also the case of the mass and pressure distribution leading the the terms of order *G* in the metric elements in Section 3. Indeed, I have solved the problem via a standard approximation that consists of averaging the matter over the entire region. For the vacuum operator ${\hat{\rho }}_{vac}\left(r,t\right)$, the problem is not too serious because that operator enters just in the mass $\hat{m}\left(r\right),$ whose definition in Equation (21) already involves an integral. However, there is a more difficult problem with the pressure operator ${\hat{p}}_{vac}$ that actually depends on the position x rather than on the radial coordinate *x* alone as in Equation (22). A plausible approximation is to average the operator over the angular variables. Then, I will use $\hat{P}\left(x\right)$, an angular average operator, rather than $\hat{p}\left(x\right),$ in Equation (22), that is

(39) $$\hat{P}\left(x\right)\to \frac{1}{4\pi }\int_{0}^{\pi }\sin\theta d\theta \int_{0}^{2\pi }d\phi \hat{p}\left(x\right)=\frac{1}{4\pi x^{2}}\int \hat{p}\left(z\right)d^{3}z\delta \left(x-z\right),$$

with $\delta \left(\right)$ being Dirac delta so that the z integral may be extended to the whole space with fair approximation.

After substituting $\hat{P}$ for $\hat{p}$ in Equation (22), we may obtain the expectation of the term of order $O(G^{2})$ belonging to the metric element ${\hat{g}}_{tt}\left(r\right)$. In order to compute the numerical value, we must introduce two new correlation functions similar to Equation (25), that is,

(40) $$\begin{array}{ccc} \frac{1}{2}\langle \psi \left|{\hat{p}}_{vac}\left(v\right)\;{\hat{p}}_{vac}\left(z\right)+\;{\hat{p}}_{vac}\left(z\right){\hat{p}}_{vac}\left(v\right)\;\right|\psi \rangle & = & C_{pp}\left(\left|v-z\right|\right),\\ \frac{1}{2}\langle \psi \left|{\hat{\rho }}_{vac}\left(v\right)\;{\hat{p}}_{vac}\left(z\right)+\;{\hat{p}}_{vac}\left(z\right){\hat{\rho }}_{vac}\left(v\right)\right|\psi \rangle & = & C_{\rho p}\left(\left|v-z\right|\right). \end{array}$$

The evaluation of the term c_3_ is as follows: taking Equations (22), (39), and (40) into account,

$$\begin{array}{ccc} c_{3} & = & 32\pi^{2}\int_{0}^{r}xdx\int_{0}^{r}ydy\langle \psi \left|\left[\hat{p}\left(x\right)\hat{p}\left(y\right)+\hat{p}\left(y\right)\hat{p}\left(x\right)\right]\right|\psi \rangle \\ & \to & 32\pi^{2}\int_{0}^{r}xdx\int_{0}^{r}ydy\langle \psi \left|{\hat{P}}_{vac}\left(x\right)\;{\hat{P}}_{vac}\left(y\right)+\;{\hat{P}}_{vac}\left(y\right){\hat{P}}_{vac}\left(x\right)\;\right|\psi \rangle \\ & = & 64\pi^{2}\int_{0}^{r}xdx\int_{0}^{r}ydy\frac{1}{16\pi^{2}x^{2}y^{2}}\int d^{3}z\delta \left(x-z\right)\int d^{3}v\delta \left(y-v\right)C_{pp}\left(\left|v-z\right|\right)\\ & = & 4\int_{z<r}z^{-1}d^{3}z\int_{v<r}v^{-1}d^{3}vC_{pp}\left(\left|v-z\right|\right), \end{array}$$

where the *x* and *y* integrals have been performed.

Now, I assume that an (approximate) equality holds similar to Equation (36). Then, I obtain

$$\begin{array}{ccc} c_{3} & = & 32\pi^{2}D_{pp}\int_{r-\gamma }^{r}zdz\int_{r}^{z+\gamma }vdv=32\pi^{2}D_{pp}\int_{r-\gamma }^{r}zdz\frac{{\left(z+\gamma \right)}^{2}-r^{2}}{2}\\ & = & 8\pi^{2}{\overset{`}{D}}_{pp}r^{2}\gamma^{2}+O\left(\gamma^{2}\right)≃8\pi^{2}r^{2}K_{pp},K_{pp}\equiv {\overset{`}{D}}_{pp}\gamma^{2}. \end{array}$$

Also, I suppose that similar approximations are valid when the density operator is combined with the pressure opertor. Thus, we may calculate $c_{4}$ and $c_{5}$ in a similar way.

$$\begin{array}{ccc} c_{4} & = & 8\pi \int_{0}^{r}dx\langle \psi \left|\left[\hat{m}\left(x\right)\hat{p}\left(x\right)+\hat{p}\left(x\right)\hat{m}\left(x\right)\right]\right|\psi \rangle \\ & \to & 8\pi \int_{0}^{r}dx\langle \psi \left|\left[\hat{m}\left(x\right)\hat{P}\left(x\right)+\hat{P}\left(x\right)\hat{m}\left(x\right)\right]\right|\psi \rangle \\ & = & 16\pi \int_{0}^{r}dx\frac{1}{4\pi x^{2}}\int d^{3}z\delta \left(x-z\right)\int_{v<x}d^{3}vC_{\rho p}\left(\left|v-z\right|\right)\\ & = & 4\int_{z<r}z^{-2}d^{3}z\int_{v<r}d^{3}vC_{\rho p}\left(\left|v-z\right|\right)\\ & = & 4D_{\rho p}\int_{r-\gamma }^{r}z^{-2}d^{3}z\int_{r}^{z+\gamma }d^{3}v\\ & = & 64\pi^{2}D_{\rho p}\int_{r-\gamma }^{r}dz\frac{{\left(z+\gamma \right)}^{3}-r^{3}}{3}=32\pi^{2}r^{2}D_{\rho p}\gamma^{2}+O\left(\gamma^{3}\right) \end{array}$$

Thus, we obtain

$$c_{4}=32\pi^{2}K_{\rho p}r^{2},K_{\rho p}\equiv D_{\rho p}.$$

$$\begin{array}{ccc} c_{5} & = & 8\pi \int_{0}^{r}x^{-2}dx\int_{0}^{r}ydy\langle \psi \left[\hat{m}\left(x\right)\hat{p}\left(y\right)+\hat{p}\left(y\right)\hat{m}\left(x\right)\right]\psi \rangle \\ & \to & 16\pi \int_{0}^{r}x^{-2}dx\int_{0}^{r}ydy\int_{0}^{x}d^{3}z\frac{1}{4\pi y^{2}}\int d^{3}v\delta \left(y-v\right)C_{\rho p}\left(\left|v-z\right|\right)\\ & = & 4\int_{0}^{r}\left(\frac{1}{z}-\frac{1}{r}\right)d^{3}z\int_{v<r}v^{-1}d^{3}vC_{\rho p}\left(\left|v-z\right|\right)\\ & = & 64\pi^{2}r^{-1}D_{p\rho }\int_{r-\gamma }^{r}\left(r-z\right)zdz\int_{r}^{z+\gamma }vdv\\ & = & 32\pi^{2}r^{-1}D_{p\rho }\int_{r-\gamma }^{r}\left(r-z\right)zdz\left[{\left(z+\gamma \right)}^{2}-r^{2}\right]=O\left(\gamma^{3}\right). \end{array}$$

Hence, the term $c_{5}$ does not contribute to order $O\left(\gamma^{2}\right).$ In summary, we have for the $g_{tt}$ element of the metric Equation (8)

(41) $$g_{tt}=1+\frac{4}{3}\pi G\rho_{mat}-\frac{8\pi G}{3}\rho_{DE}r^{2}+8\pi^{2}G^{2}r^{2}\left(K+K_{pp}+4K_{\rho p}\right).$$

It is plausible that the quantities *K* and $K_{pp}$ are both positive but $K_{\rho P}$ is negative. In fact, we may assume that in quantum vacuum fluctuations the pressure acts with a sign opposite to the mass density, in agreement with the Lorentz invariant vacuum equation of state p=−ρ. This suggests identifying

(42) $$K_{pp}=K,K_{\rho p}=-K$$

whence we obtain, taking Equations (23) and (38),

(43) $$g_{rr}=1+\frac{8\pi G}{3}\left(\rho_{B}\left(t\right)+\rho_{DM}\left(t\right)+\rho_{DE}\right)r^{2}+16\pi^{2}G^{2}Kr^{2}.$$

Similarly, from Equations (41) and (42) we obtain

(44) $$g_{tt}=1+\frac{8\pi G}{3}\left(\frac{1}{2}\rho_{B}\left(t\right)+\frac{1}{2}\rho_{DM}\left(t\right)-\rho_{DE}\right)r^{2}-16\pi^{2}G^{2}Kr^{2}.$$

These results reproduce the standard ones Equation (11) plus a correction due to the quantum vacuum fluctuations (i.e., the last term in Equations (43) and (44)).

## 4. Results and Discussion

The main result of this article is that Equations (43) and (44) should be substituted for the standard Equation (11). Then, the following should be substituted for Equation (1)

(45) $$\begin{array}{ccc} \rho_{DE}+6\pi GK & ≃ & \left(6.0\pm 0.2\right)\times 10^{-27}\;\mathrm{kg}/m^{3}\\ & \Rightarrow & 0<K≲\frac{\left(6.0\pm 0.2\right)\times 10^{-27}\;\mathrm{kg}/m^{3}}{6\pi G}. \end{array}$$

The conclusion is that either the acceleration in the expansion of the universe is due to the quantum vacuum (if the latter inequality is really an equality) or the vacuum gives just a contribution to be added to the effect of a dark energy. In the former case, the value of the parameter K would be following

(46) $$K\equiv D\gamma^{2}=\frac{\rho_{DE}c^{2}}{6\pi G}≃0.42\;{\mathrm{kg}}^{2}/m^{4},\sqrt{K}≃0.65\;\mathrm{kg}/m^{2}.$$

Taking Equations (31)–(34) into account, the quantity $\sqrt{K}$ may be seen as the product of the typical mass density of the vacuum fluctuations $\sqrt{D}$ times its typical correlation length γ. It is fitting that the value of $\sqrt{K}$, Equation (46), is not too far from the product of the typical nuclear density, 2.3 × 10^17^ kg/m^3^, times a typical nuclear radius, about 10^−15^ m. For instance, if the correlation length of the vacuum energy density was 10^−11^ m, a typical atomic distance, then the fluctuation of the density would be about 10^−7^ times the nuclear density.

In summary, our work does not prove that quantum vacuum fluctuations are a valid alternative to dark energy. But it does show that such fluctuations give rise to a contribution with qualitative effects similar to those of the dark energy.

## Data Availability

Data are contained within the article.

## Appendix A. A Note on Interpretation

In the calculation of the present paper I have not attempted any interpretation, but the treatment has followed the standard quantum formalism. However, a few comments on interpretation are in order.

After one century of quantum mechanics, there is no agreement about the interpretation of the theory [16], in particular about the real meaning of the “quantum probability”. The standard wisdom is that quantum probabilities are dramatically different from the common probabilities used in so many areas, from economics or biology to classical statistical mechanics [17]. Then, there are two types of probability in quantum theory, one in the measurement, the other type in the definition of mixed states. The latter are similar to the common probabilities above mentioned [17], and they may be associated with incomplete information.

In the measurement of the properties of a pure state, several different results may be obtained with a definite *probability* each. These probabilities are *not* attributed to incomplete information about the state of the system, which is assumed to be pure. The common opinion is that they appear due to a lack of causality of the physical laws, a strange assumption indeed. I support the view that there are “hidden variables” that might determine the results of the measurements. If this is the case, the probabilities involved are also standard [17], that is, no specific “quantum probabilities” exist. The current wisdom is that suitable hidden variables, that is, local, are not possible [18,19,20]. See, however, [21,22]. The described situation also applies to the particular case of the “vacuum state”. This state is believed to be pure, but vacuum fluctuations exist that are also believed to correspond to the peculiar “quantum probabilities”.
